# His Family Doctors

**Published:** 1900-05

**Authors:** 


### His Family Doctors.

When the new boy got into the school room he was, of course, pestered with numerous questions by the other scholars as to his name, his parent's profession, the amount of his pocket-money, and various other matters about which boys are curious.

"Who's your family doctor?" asked a big lad.

"Ain't got none!" was the prompt if ungrammatical reply.

"How jolly!" responded the questioner. "Why, you didn't have no medicine to take!"

"Didn't I?" was the sarcastic reply. "That's all you know. Why, my father's an homeopath, mother's an allopath, my sister Maggie joined the ambulance, grandfather believes in resustication from drowning, grandmother goes in for every quack medicine that's advertised, my uncle Sandy's a horse-doctor, and—" with a pathetic tone—"they all of them experimented on me!"

That boy got the sympathy he desired.

